# Endoscopic duodenal stenting is efficient, but has higher rate of reoperations than gastrojejunostomy in palliative treatment for gastric outlet obstruction

**DOI:** 10.1007/s00423-022-02565-x

**Published:** 2022-06-01

**Authors:** Matti Laitamäki, Tuula Tyrväinen, Juho T. Lehto, Johanna Laukkarinen, Mika Ukkonen

**Affiliations:** 1 Department of Gastroenterology and Alimentary Tract Surgery, Tampere University Hospital, Kuntokatu 2, 33520 ElämänaukioTampere, Finland; 2 Faculty of Medicine and Health Technology, Tampere University, Tampere, Finland; 3 Palliative Care Centre and Department of Oncology, Tampere University Hospital, Tampere, Finland

**Keywords:** Palliative care, Palliative surgery, Gastric outlet obstruction, Duodenal stenting, Gastrojejunostomy

## Abstract

**Background:**

Surgical gastrojejunostomy has traditionally been the palliative treatment of choice for patients with advanced malignancies and gastric outlet obstruction syndrome. Recently, palliative endoscopic duodenal stenting has increased in popularity. We report outcomes after gastrojejunostomy and duodenal stenting when used for palliative indications.

**Methods:**

Consecutive patients undergoing palliative gastrojejunostomy or palliative endoscopic duodenal stenting in a Finnish tertiary referral center between January 2015 and December 2020 were included. The postoperative outcomes of these two palliative interventions were compared. The main outcome measures were mortality and morbidity, rate of reoperations, postoperative oral intake ability, and length of hospital stay.

**Results:**

A total of 88 patients, 46 (52%) patients underwent palliative gastrojejunostomy and 42 (48%) duodenal stenting. All patients had malignant disease, most typically hepatopancreatic cancer. Nineteen (44%) patients in duodenal stenting group and 4 (8.7%) patients in gastrojejunostomy group required subsequent interventions due to persisting or progressing symptoms (*p* < 0.001). Median delay until first oral intake was 2 days (1–24) after gastrojejunostomy and 0 days (0–3) after stenting (*p* < 0.001). Postoperative morbidity was 30% after gastrojejunostomy and 45% after stenting (*p* < 0.001). Median length of hospital stay was 7 days (1–27) after surgery and 5 days (0–20) after endoscopy (*p* < 0.001).

**Conclusions:**

Patients undergoing endoscopic duodenal stenting are more able to initiate rapid oral intake and have shorter hospital stay. On the other hand, there are significantly more reoperations in stenting group. If the patient’s life expectancy is short, we recommend stenting, but for patients whose life expectancy is longer, gastrojejunostomy could be a better procedure, for the reasons mentioned above.

## Introduction

Gastric outlet obstruction (GOO) is a constrictive process of the duodenum often associated with advanced gastrointestinal malignancies. [1, 2] Open surgical gastrojejunostomy (GJ) has traditionally been the treatment of choice for patients with advanced malignancies. Although considered a relatively simple procedure, it is associated with significant morbidity and mortality. Less invasive techniques, such as laparoscopic GJ and endoscopic duodenal stenting with self-expanding metal stents (SEMS), have recently increased in popularity. [1–3] While earlier studies have reported lower morbidity associated with endoscopy, long-term functional outcomes are reportedly better after surgery [4, 5]. As patients with advanced and incurable cancer are often particularly frail, the advantages of endoscopy have included that endoscopic procedures can be performed in sedation, in contrast to GJ, which requires general anesthesia. [6]

While some earlier studies have been presented, there is only scant data comparing outcomes of different treatment modalities. Consequently, the aim of this study was to compare the short- and long-term outcomes of GJ and stenting in the palliative care of GOO. [1, 4, 7, 8].

## Materials and methods

**Fig. 1:**
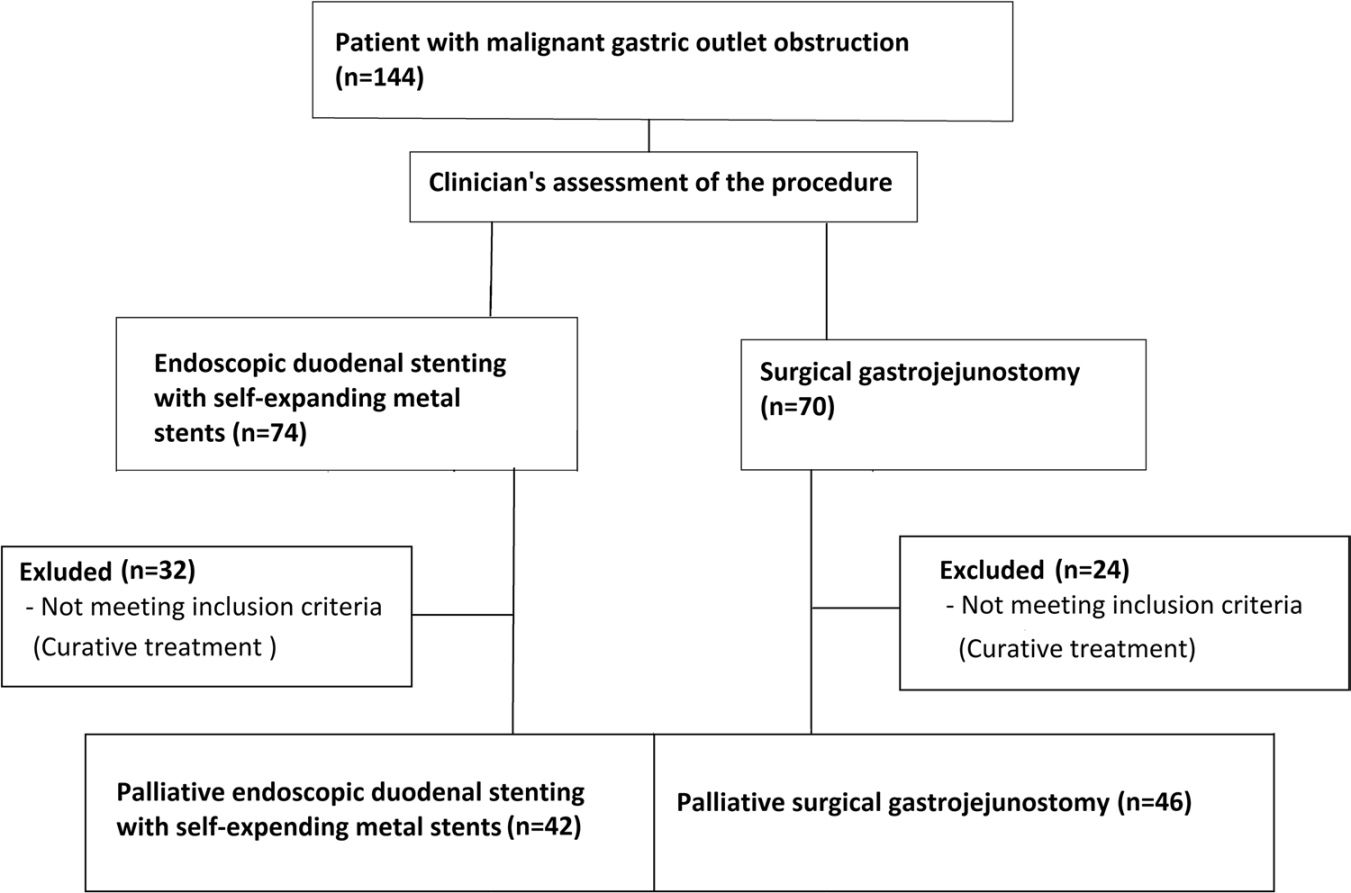
Flowchart of the study

The study is based on retrospective data. Each consecutive patient who underwent palliative GJ or SEMS for malignant GOO in Tampere University Hospital, Finland from January 1, 2015 to December 31, 2020 was included in study**.** A total of 74 endoscopic duodenal stentings were performed during follow-up, of which 32 were excluded because operation sought curative and in 42 were palliative procedures. At the same time, 70 gastrojejunostomies were performed at Tampere University Hospital, of which 46 were palliative procedures as shown in Fig. 1. Patients’ medical records were reviewed. Only those undergoing the procedure for a palliative indication were included. Patients receiving neoadjuvant therapies after stenting or patients with GOO due to benign causes, such as pancreatitis, were excluded. The type of procedure was selected based on the judgment of an experienced clinician.

Patients were identified by retrieving all cases associated with the Nordic Medico-Statistical Committee classification of surgical procedures (version 1.13) codes. The study population included all patients requiring surgical or endoscopic treatment for malignant GOO in a catchment area of over 500,000 inhabitants as well as some referred patients, when the hospital catchment area exceed one million inhabitants.

Patient data was collected from the Tampere University Hospital surgical database and the medical records. Patient characteristics were recorded, and included age, sex, comorbidities, type of malignancy, preoperative functional ability, preoperative Gastric Outlet Obstruction Scoring System (GOOSS), and indication for the procedure. Main outcomes were postoperative morbidity (according to Clavien-Dindo classification of surgical complications) [9], reoperations, postoperative GOOSS, postoperative oral intake ability, postoperative length of hospital stay, follow-up treatment location, and mortality. GOOSS is one of the most frequently used scoring systems for patients with GOO. GOOSS was originally developed by Adler in 2002. Its ranges 0–3 (0: no oral intake at all, 1: liquids oral intake, 2: soft foods oral intake, 3: normal food oral intake). [10]

Patients underwent either surgery or endoscopic procedures. Both laparoscopic and open surgical techniques were used. GJ was performed by using side-to-side loop anastomosis technique. For endoscopic procedures, the study hospital offered two different self-expanding stent options for duodenal stenting, namely uncovered metal stents (Olympus Hanarostent®) and duodenal stents (Boston scientific Wallflex®). Several size options were available for both stents, which were selected according to the clinician’s assessment.

Statistical analyses were performed using SPSS Statistics version 22 for Windows (IBM Corp, Armonk, NY, USA). Summary measurements were expressed as means with standard deviations or as medians with minimum and maximum values unless otherwise stated. Continuous variables were analyzed using Student’s *t*-test or Mann–Whitney *U*-test, the latter for non-normally distributed data. Chi-square or Fisher’s exact test was used for categorical variables. Two-tailed *P* values were reported and a *P* value < 0.05 was deemed statistically significant.

The study was conducted according to the requirements of the Helsinki declaration. In compliance with the principles of the local ethics committee, exemption from consent was obtained as the data had already been collected for clinical purposes.

## Results

Forty-six patients (24 females, median age 66 years [47–92]) underwent GJ and 42 patients (16 females, median age 72 years [37–95]) SEMS placement. Median follow-up time was 76 days (3–972). Demographic characteristics were practically similar. Most of patients were diagnosed with hepatobiliary (*n* = 10 in the GJ group and *n* = 2 in the SEMS group) or pancreatic cancer (*n* = 16 in the GJ group and *n* = 25 in SEMS group) (59% in the GJ group and 62% in the SEMS group). Other malignities were in the SEMS group and in the GJ group: gastric (*n* = 7 and *n* = 4), duodenal (*n* = 3 and *n* = 6), colorectal (*n* = 1 and *n* = 3), other (*n* = 5 and *n* = 6). The share of patients with locally advanced cancer was 46% in the GJ group and 21% in the SEMS group (*p* = 0.006) and 52% of GJ group patients and 60% SEMS group patients had distant metastases or peritoneal carcinosis (*p* = 0.006). Twenty-four percent of the GJ group and 33% of the SEMS group had peritoneal carcinosis (*p* = 0.328). Table 1 shows the main characteristics of the study population.

**Table 1:** Demographic data of the study population

Variable	Gastrojejunostomy	Duodenal stenting	
Population *n* (%)	46 (52)	42 (48)	
Age, median (min–max)	66 (47–92)	72 (37–95)	0.085
Female, *n* (%)	24 (52)	16 (38)	0.185
BMI, median (min–max)	24 (17–57)	26 (18–44)	0.072
Smoking, *n* (%)	17 (37)	10 (24)	0.182
Comorbidities, *n* (%)	41 (89)	38 (91)	
Diabetes	17 (37)	14 (33)	0.144
Hypertension	31 (67)	25 (60)	0.443
Heart failure	1 (2.2)	4 (9.5)	0.137
COPD	4 (8.7)	3 (7.1)	0.788
Coronary artery disease	8 (17)	4 (9.5)	0.283
Hypothyroidism	4 (8.7)	5 (12)	0.620
Atrial fibrillation	3 (6.5)	11 (26)	0.012
Malignancy, *n* (%)			0.759
Hepatopancreatic	27 (59)	26 (62)	
Other	19 (41)	16 (38)	
Preoperative functional ability, *n* (%)			0.342
Independent in daily activities	31 (67)	23 (55)	
Partially dependent in daily activities	13 (28)	18 (43)	
Totally dependent in daily activities	2 (4.3)	1 (2.4)	
Advanced cancers, *n* (%)	45 (98)	34 (81)	0.006
Locally advanced	21 (46)	9 (21)	
Metastatic cancers	24 (52)	25 (60)	
Peritoneal carcinosis, *n* (%)	11 (24)	14 (33)	0.328
Ascites, *n* (%)	6 (13)	15 (36)	0.013
Preoperative chemotherapy	9 (20)	13 (31)	0.218
Steroid drugs preoperatively, *n* (%)	5 (11)	13 (31)	0.020
ASA physiological status, *n* (%)			
1–2	4 (8.7)	-	
3–5	42 (91)	-	
Preoperative vomiting, *n* (%)	36 (78)	33 (79)	0.972
Nasogastric tube preoperatively, *n* (%)	29 (63)	20 (49)	0.181
Laparoscopic operation, *n* (%)	6 (13)	-	
Preoperative GOOSS, *n* (%)			0.031
0	27 (59)	22 (52)	
1	7 (15)	12 (29)	
2	5 (12)	1 (2.2)	
3	3 (7.1)	11 (24)	
Stent model, *n* (%)			0.534
Uncovered metal stent	-	29 (71)	
Fully covered metal stent	-	12 (29)	

*COPD*, chronic obstructive pulmonary disease; *ASA physiological status*, American Society of Anesthesiologists classification; *Preoperative GOOSS*, preoperative Gastric Outlet Obstruction Scoring System

Seventy-one percent of SEMS patients received uncovered metal stents and 29% fully covered metal stents. Thirteen of GJ group had undergone stenting attempt, the most typical reason for failure was complete stenosis, i.e., stenting was not technically feasible. The mean interval from the first stenting to the second procedure was 9 days (0–313). A typical reason for the renewed stenting was gastric outlet obstruction. Two patients suffered from stenting associated perforation and required emergency laparotomy and gastrojejunostomy immediately after the operation.

Only one patient required postoperative ICU care. Nineteen patients (45%) in the SEMS group and four (8.7%) in GJ underwent reoperation. In the SEMS group, the most typical reoperation was re-stenting (21%) or gastrojejunostomy (21%). Most typical reason for reoperation was stent obstruction as the disease progressed or problems with gastric emptying even stent was open. Morbidity was 30% in the GJ group and 45% in the SEMS group (*p* < 0.001). In-hospital mortality was 6.5% in the GJ group and 2.4 in SEMS group (*p* = 0.113). Short-term hospital readmissions (in 3 months after procedure) were 7 (15%) in GJ group and 17 (41%) in SEMS group (*p* = 0.003). There was no significant difference in postoperative GOOSS in the study population (*p* = 0.899). Among GJ patients, the median delay to feeding initiation with liquids was 2 days (1–24) and less than 1 day (0–3) in the SEMS group. Patients in the GJ group received normal food after 6.5 days (1–10) and after 2 days (0–5) in SEMS group. Table 2 show postoperative outcomes.

**Table 2:** Postoperative outcomes

Variable	Gastrojejunostomy		Duodenal stenting	
Admission to ICU	0		1 (2.4)	0.293
Morbidity, *n* (%)	14 (30)		19 (45)	< 0.001
Minor (CD I–II)	8 (17)		0	
Major (CD III–IV)	6 (13)		19 (45)	
Reoperation (%)	4 (8.7)		19 (44)	< 0.001
Duodenal stenting	0		9 (21)	
Gastrojejunostomy	0		9 (21)	
Other	4 (8.7)		1 (2.4)	
In-hospital mortality, *n* (%)	3 (6.5)		1 (2.4)	0.352
Length of hospital stay, days (min–max)	7 (1–27)		5 (0–20)	< 0.001
Functional ability, *n* (%)				0.357
Independent in daily activities	12 (28)		9 (22)	
Partially dependent in daily activities	24 (56)		20 (49)	
Totally dependent in daily activities	7 (16)		12 (29)	
Short-term hospital readmission, *n* (%)	7(15)		17 (41)	0.003
Location for follow-up treatment, *n* (%)				0.959
Home, independently	9 (21)		8 (20)	
Home, with home care	5 (12)		4 (9.8)	
Residential care	0		1 (2.4)	
Community Hospital	9 (21)		12 (29)	
Secondary or tertiary care hospital	18 (42)		4 (32)	
Palliative care ward	2 (4.7)		3 (7.3)	
Postoperative survival, days, (median, min–max)	108 (3–972)		50 (9–597)	0.016
Mortality rates, *n* (%)	43 (94)		42 (100)	0.092
14 days	4 (9.5)		7 (17)	0.332
30 days	10 (24)		14 (33)	0.334
90 days	19 (45)		32 (76)	0.004
1 year	34 (81)		41 (98)	0.014
Postoperative GOOSS, *n* (%)				0.899
0	4 (9.3)		6 (15)	
1	9 (21)		8 (20)	
2	24 (56)		22 (54)	
3	6 (14)		5 (12)	
Days to oral intake median, (min–max)				
Liquids	2 (1–24) *n* = 40	0 (0–3) *n* = 33		< 0.001
Soft foods	4 (1–26) *n* = 29	2 (1–6) *n* = 25		< 0.001
Normal food	6.5 (1–10) *n* = 6	2 (0–5) *n* = 6		0.074
Days with nasogastric tube, median (min–max)	2 (0–10) *n* = 40		1 (0–6) *n* = 7	0.068
Postoperative vomiting, *n* (%)	12 (28)		13 (35)	0.487

*CD*, Clavien-Dindo classification; *GOOSS*, Gastric Outlet Obstruction Scoring System

**Fig. 2:**
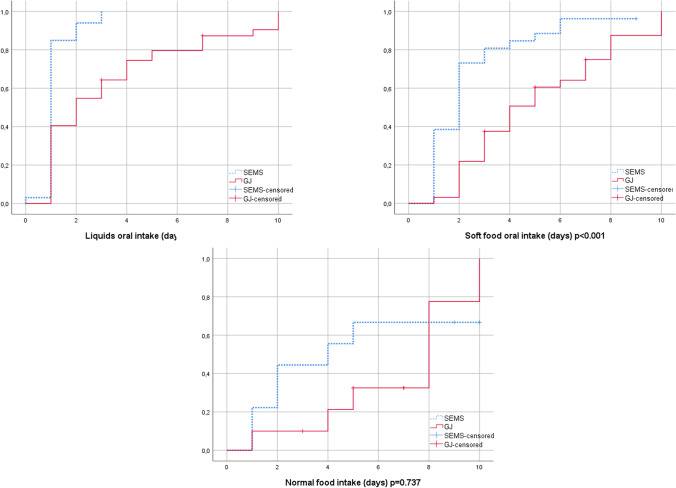
Oral intake

Times to postoperative oral intake of the patient groups by Kaplan–Meier graphs are presented in Fig. 2. In the SEMS group, oral intake of fluids (*p* < 0.001), soft foods (*p* < 0.001), and normal food (*p* = 0.737) was faster than in the GJ group.

**Fig. 3:**
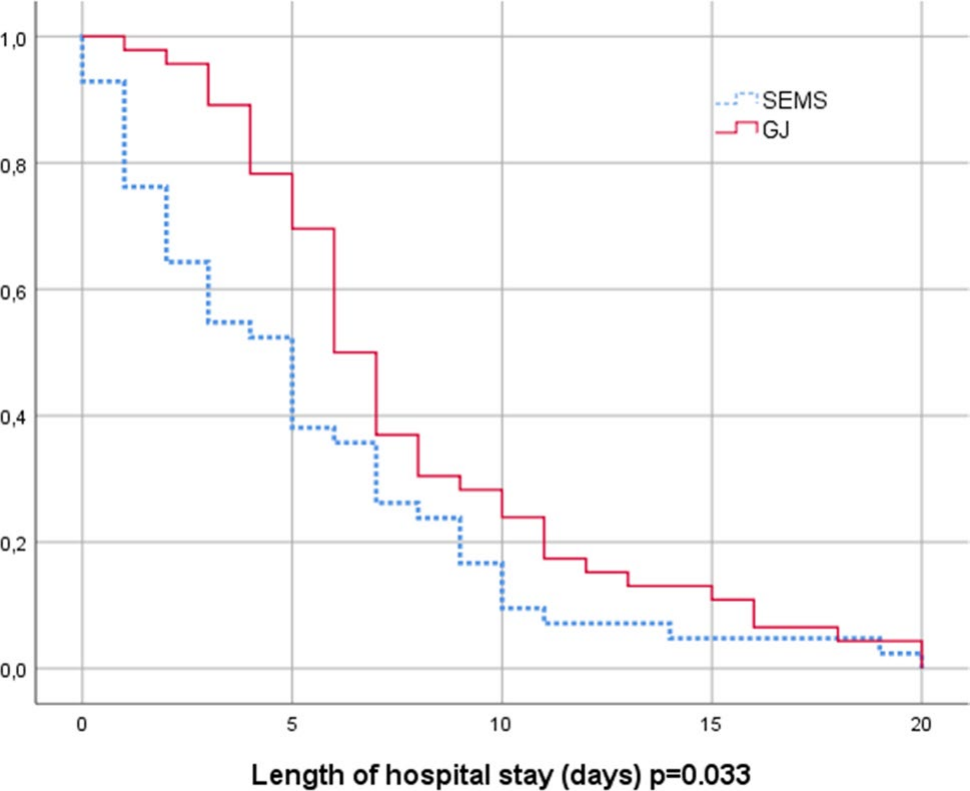
Length of hospital stay

**Fig. 4:**
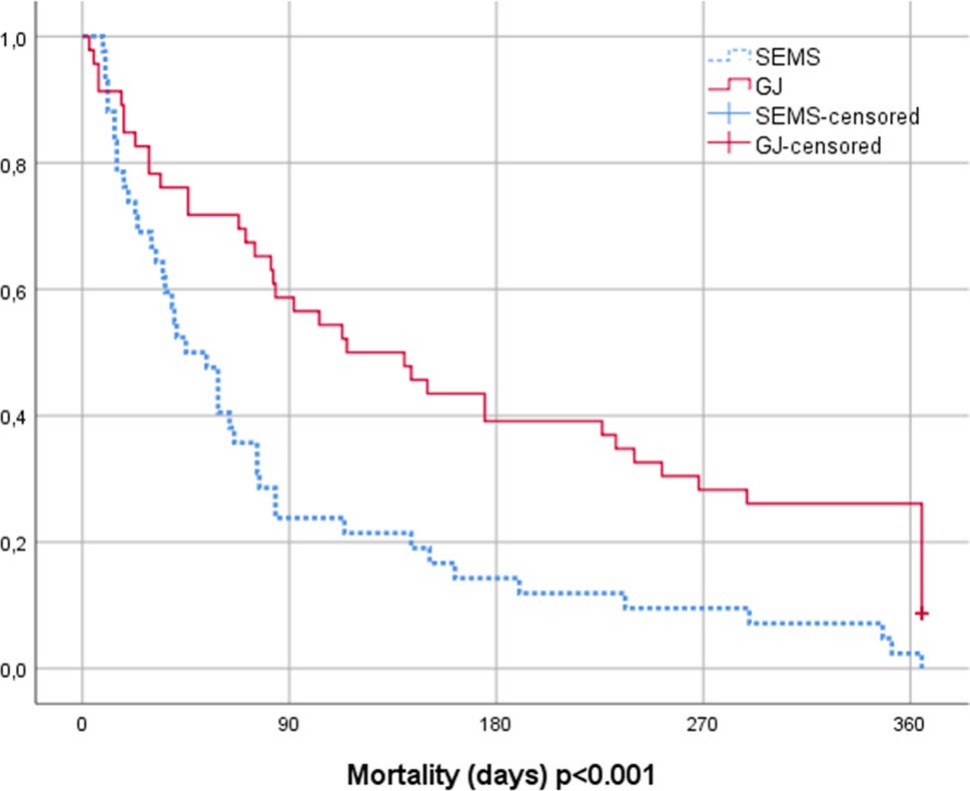
Mortality

Kaplan–Meier curves illustrating lengths of hospital stay are shown in Fig. 3 and mortality in Fig. 4. Median length of stay in the GJ group was 7 days (1–27) and in the SEMS group 5 days (0–20), *p* = 0.002. The overall mortality rate during follow-up was 94% in the GJ group and 100% in the SEMS group (*p* = 0.092). Median survival in the GJ group was 108 days (3–972) and 50 days (9–597) in the SEMS group (*p* = 0.016).

## Discussion

This study compared the two most commonly used methods for palliative relief of malignant GOO. Patients receiving SEMS were able to initiate oral intake more rapidly and had shorter hospital stay than those receiving with GJ but had higher rates of reoperations and mortality.

The goals of palliative care differ from those of curative care. Palliative care aims to prevent and alleviate physical, emotional, and mental suffering among patients dealing with advanced and incurable illness. [11–13] Such patients have limited expected life span, and it is easy to understand that rapid recovery, including initiation of oral intake and short hospital stay, are of the utmost importance. Our results were consistent with earlier findings; patients in the SEMS group recovered more rapidly and were able to initiate oral intake faster than those undergoing surgery. Stent insertion is a less invasive procedure, which may explain the faster alleviation of symptoms. [4, 6–8, 14] While symptoms were relieved in the majority of patients, a significant portion of our patients required further care at other health care facilities or in residential care. Only a few patients were able to return to their own homes. There was no significant difference between the GJ and SEMS groups in terms of follow-up care. Other important goals of palliative care include low morbidity. Patients are often in poor clinical condition due to multiple days of reduced food intake and weight loss. SEMS group had higher rate of reoperations. Complications were more common after SEMS, but almost every complication that lead to reoperation was related to slowed gastric emptying. Two patients underwent emergency surgery due to stent-associated perforation immediately after stenting and both were treated surgically with gastrojejunostomy. This is comparable to major early complications reported in earlier publications. However, in our study population, there was no bleeding after stenting, which is another typical early major complication. [5, 6, 15] There were no long-term complications associated with stenting, i.e., there were no cases with stent migration or late perforation. While mortality was significant in both patient populations, there were no procedure-associated deaths. Short-term readmissions (max 3 months) were more common among patients with SEMS.

The data were collected in a single high-volume tertiary care center with experienced endoscopists and surgeons performing all procedures. The most significant strength of the present study was the inclusion of all patients undergoing palliative surgery within the second largest hospital district in Finland. The data was comprehensive and included follow-up data on all patients. In our hospital, stents were placed in with the patient under sedation. Patients did not receive general anesthesia unless it was absolutely necessary (e.g., due to fear related to endoscopy or co-operation difficulties). Therefore, we consider patients with more advanced disease and significant co-existing conditions might have undergone stenting instead of possible surgery. This may explain the poorer long-term outcome among SEMS patients, but as noted there were no procedure-related deaths. The relatively short survival among patients with advanced and incurable cancer is not exceptionally dismal. The biggest weakness in this study is the retrospective data, where the clinician’s view of patients is likely to cause selection bias.

## Conclusion

The results of our study are similar to those of earlier studies. [1, 7] Patients undergoing endoscopic duodenal stenting are more able to initiate rapid oral intake and have shorter hospital stay. On the other hand, there are significantly more reoperations in stenting group. If the patient’s life expectancy is short, we recommend stenting, but for patients whose life expectancy is longer, gastrojejunostomy could be a better procedure, for the reasons mentioned above. However, further qualitative research on the subject is needed, especially as new and interesting treatment options such as endoscopic ultrasound–guided gastrojejunostomy have been introduced for clinical use. Our study group have plans to conduct an RTC study on this challenging palliative topic in the future.
